# Toward Routine Minimally Invasive Ventricular Septal Defect Closure *Via* Right Lateral Minithoracotomy

**DOI:** 10.3389/fped.2021.708203

**Published:** 2021-08-10

**Authors:** Selim Aydin, Bahar Temur, Serdar Basgoze, Fusun Guzelmeric, Osman Guvenc, Ersin Erek

**Affiliations:** ^1^Department of Cardiovascular Surgery, Faculty of Medicine, Acibadem Mehmet Ali Aydinlar University, Istanbul, Turkey; ^2^Department of Anesthesiology, Atakent Hospital, Acibadem Mehmet Ali Aydinlar University, Istanbul, Turkey; ^3^Department of Pediatric Cardiolog, Atakent Hospital, Acibadem Mehmet Ali Aydinlar University, Istanbul, Turkey

**Keywords:** minimally invasive, congenital heart surgery, ventricular septal defect, minithoracotomy, pulmonary stenosis

## Abstract

**Background:** Improving the surgical results and recent advancement of transcatheter techniques for closure of ventricular septal defect (VSD) increased the demand for minimally invasive approaches. In this study, we analyzed the results of the patients who underwent VSD closure with right lateral minithoracotomy (RLMT).

**Methods:** Between September 2014 and February 2021, 24 patients underwent minimally invasive VSD closure with RLMT. The median age of the patients was 16 months (range, 4-84 months). Fifteen patients (62.5%) were female. The median weight of the patients was 9.75 kg (range, 4.6-30 kg). The types of VSD were perimembranous in 19 patients, subaortic in three patients, inlet in one patient, and subpulmonic in one patient. Five patients had low-lying pulmonary stenosis in addition to VSD.

**Results:** No perioperative death or major complication occurred during follow-up. All defects were repaired through RLMT. The median cardiopulmonary bypass time was 81 min (range, 44-163 min), and the aortic cross-clamp time was 65 min (range, 33-131 min). The median hospital stay was 6 days (range, 5-21 days). One patient had minimal (2 mm) residual left-to-right shunt. All families were satisfied with the cosmetic results during the follow-up.

**Conclusions:** The RLMT method is a safe and effective alternative to standard median sternotomy for VSD closure and can be performed with favorable cosmetic and clinical results.

## Introduction

Ventricular septal defect (VSD) is the most common congenital heart defect in children. Evolving surgical techniques and intensive care facilities significantly reduced the mortality and morbidity rates of surgery for VSD closure, which enables normal life expectancy and quality of life. Although median sternotomy is still the most preferred approach in many centers, a large incision scar may cause cosmetic and psychosocial problems (1). Improving the surgical results and recent advancement of transcatheter techniques for closure of VSD increased the demand for minimally invasive approaches. Unsuitability or failure of transcatheter VSD closure switches the condition from no incision to full sternotomy, and it is usually frustrating for the family and the patients. Using minimally invasive techniques for congenital heart diseases is very rare and usually limited to atrial septal defect (ASD) closure. Various alternative approaches have been described to improve cosmetic results, such as partial sternotomy and right antero- or posterolateral thoracotomy (2). However, they have been found to be suboptimal because of visibility of the scar, risk for thorax deformity, or breast asymmetry, especially when performed in prepubescent girls (3). Nevertheless, most of the patients who need surgical VSD closure are small infants for whom minimally invasive techniques seem more difficult to perform due to small thoracic cavity and cardiac structures.

**Figure 1:**
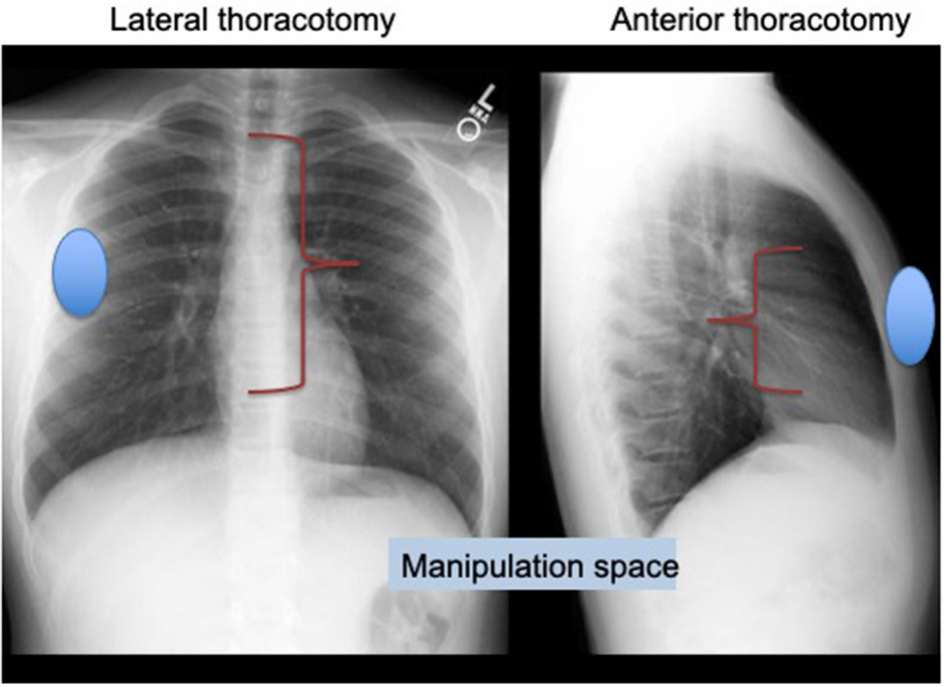
Lateral thoracotomy provides more manipulation space than anterolateral thoracotomy approach.

We have been using right lateral minithoracotomy (RLMT) approach with central cannulation for more than 20 years. We believe that the cosmetic results are better and there is no risk for breast asymmetry because the incision lies quite far from the breast tissue and is hardly visible on the anterior chest, staying under the right armpit. It provides more manipulation space than the anterolateral thoracotomy approach (Figure 1). Our surgical experience started with ASD closure and evolved to partial atrioventricular septal defect (AVSD), partial pulmonary venous return anomaly, VSD closure, and recently VSD and pulmonary stenosis (PS) repair in small infants with the same incision. In this study, we reported the results of the patients who underwent VSD closure with RLMT.

## Patients and Methods

**Figure 2:**
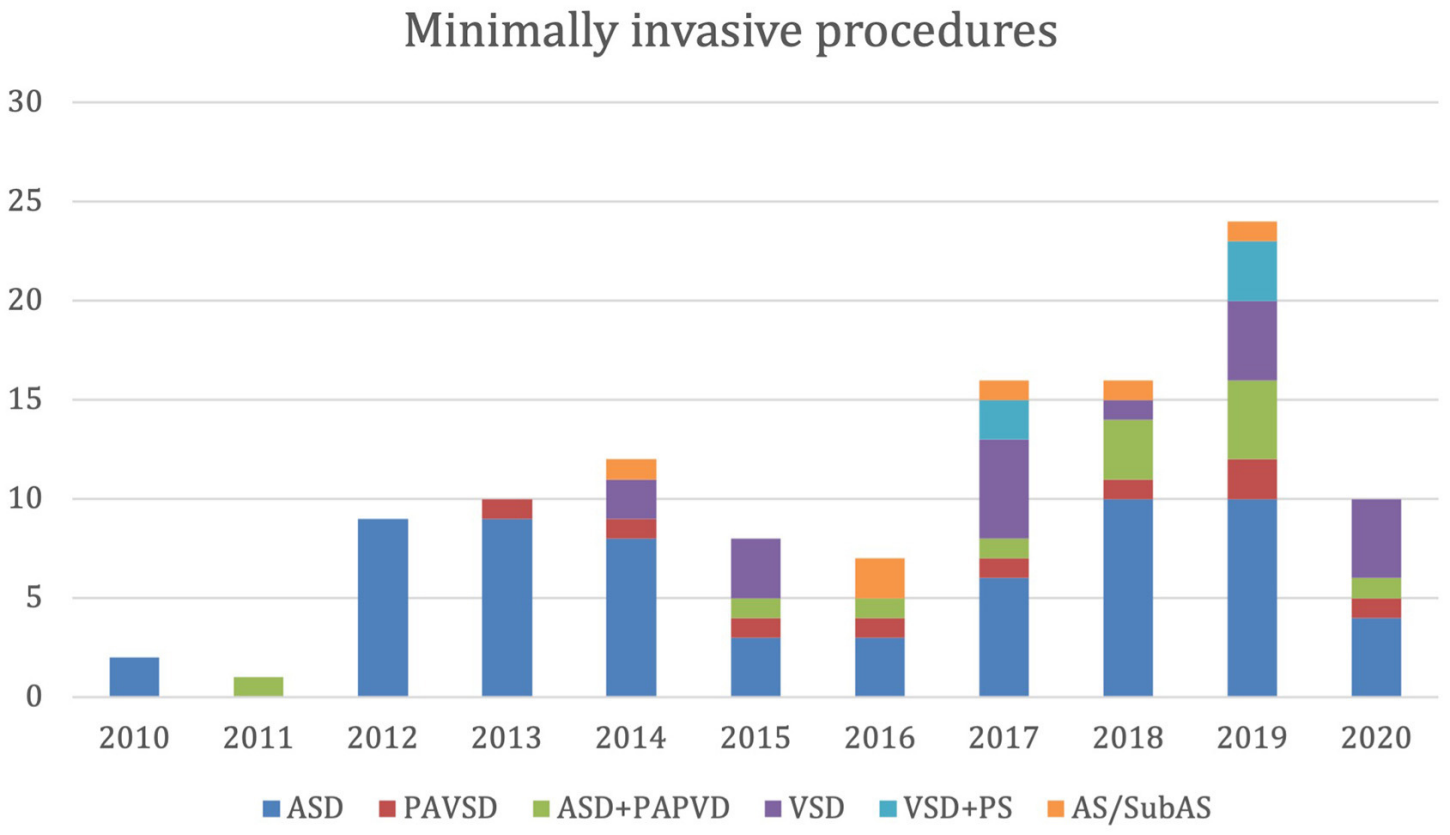
Number and diversity of the minimally invasive procedures.

Our retrospective study received ATADEK-2021/07 number 2020-07/31 Ethics Committee approval on July 4, 2021. Between October 2010 and February 2021, 116 patients underwent minimally invasive congenital heart surgery. All patients had RMLT incision, except six patients who had superior J sternotomy due to aortic or subaortic stenosis repair. The number and the diversity of the procedures are demonstrated in Figure 2. We have started minimally invasive VSD closure in 2014 and VSD and low-lying PS repair in 2017 with increasing complexity. Among all patients, 19 had VSD closure and five had VSD-PS repair that constitute the study group. During this period, VSD repair was performed with median sternotomy in 225 patients, with one mortality. Although it is technically more difficult to repair subpulmonic and multiple VSDs, any type of VSD can be repaired with the RLMT approach. We performed the RLMT approach for children over 5 kg and according to family preference. However, with our increasing experience, we started to perform RLMT to patients under 5 kg as in one patient in the article. The median age of the patients was 16 months (range, 4-84 months). Fifteen patients (62.5%) were female. The median weight of the patients was 9.75 kg (range, 4.6-30 kg). Twelve patients (50%) were under 10 kg. The types of VSD were perimembranous in 19 patients, subaortic in three patients, inlet in one patient, and subpulmonic in one patient. Five patients had a low-lying PS in addition to VSD. The size of the VSDs was large in 10 patients (41.6%). Others had moderate-sized VSDs. None of the patients were suitable for transcatheter VSD closure, or some had a failed attempt.

### Operative Technique

**Figure 3:**
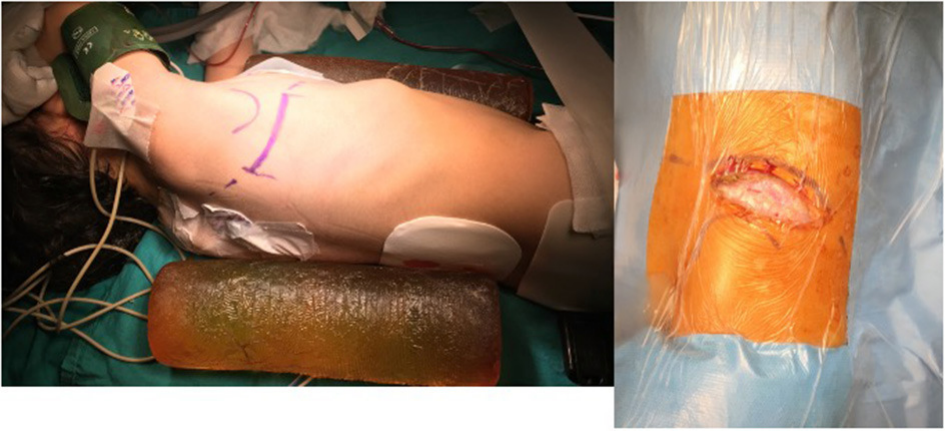
Lateral minithoracotomy incision.

**Figure 4:**
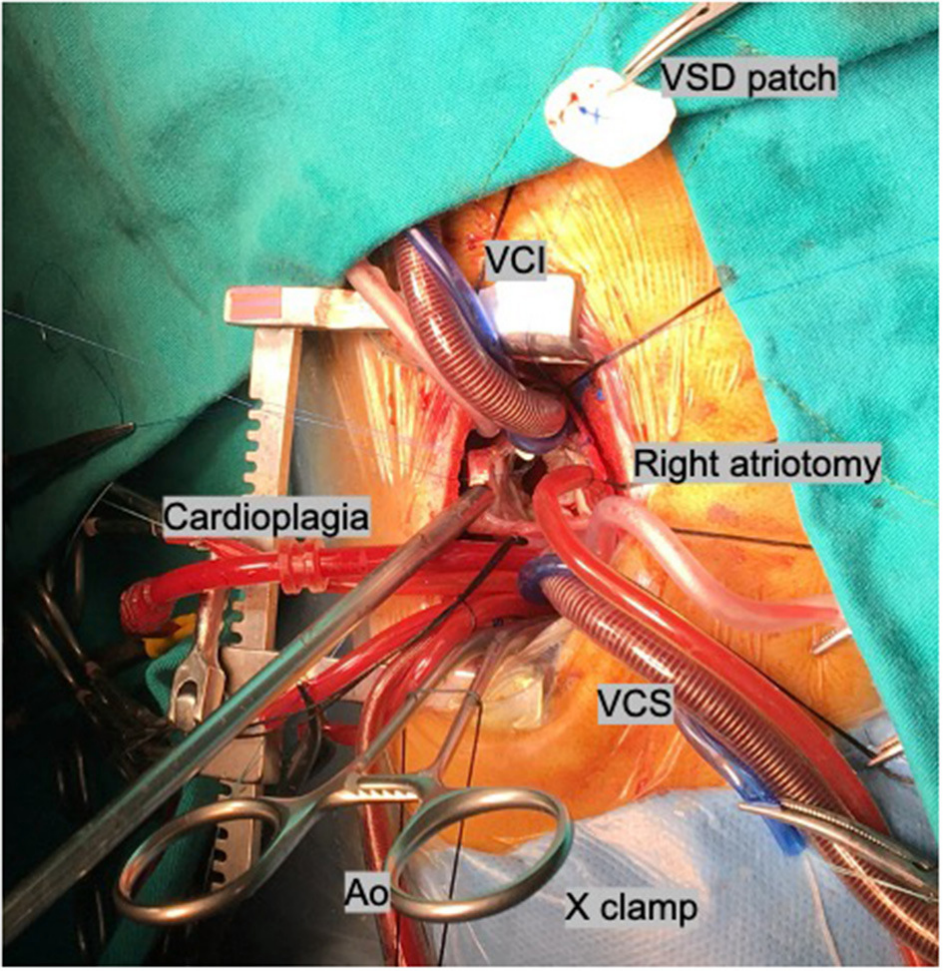
Central cannulation. Ao, aortic cannula; VCS, vena cava superior; VCI, vena cava inferior.

**Figure 5:**
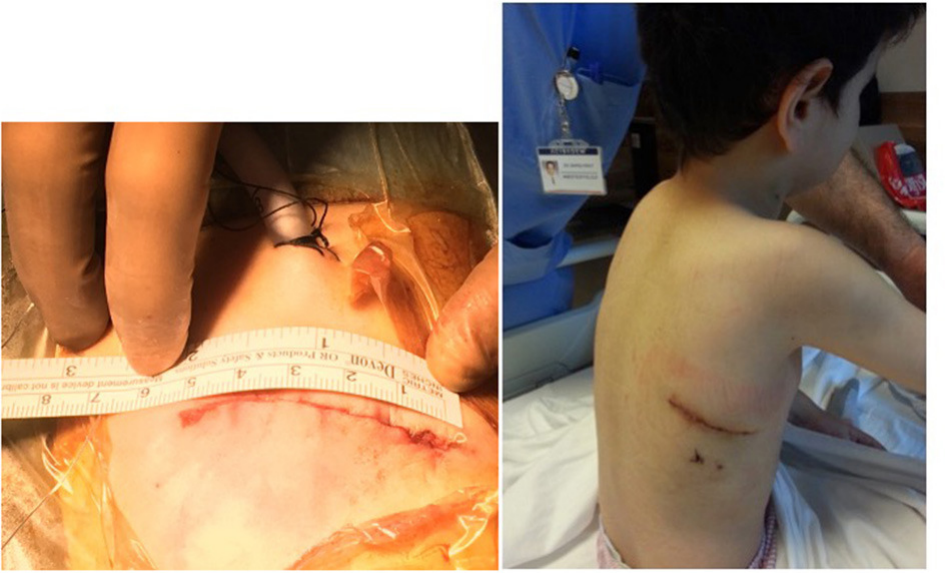
Right minithoracotomy incision length and scar.

After general anesthesia, a pediatric transesophagial echocardiographic probe was inserted. Defibrillation pads were stuck to the anterior and posterior parts of the left hemithorax. The patients were positioned in 90° left lateral decubitus position, and the right arm was put over the head in a natural position. An ~5–7-cm skin incision was performed obliquely between the anterior and posterior axillary lines on the sixth intercostal space, which is the lower end of the scapula (Figure 3). Afterwards, the scapula was lifted with retractors, and the thoracic cavity was entered through the fourth intercostal space, where easier access to the heart and main vessels was provided. A rib spreader was used in all patients. The pericardium was opened 2 cm anterior to the phrenic nerve, superiorly to the pericardial reflection and inferiorly to the diaphragm. The pericardium was hung with retraction silk stitches, and a wet sponge was placed over the right lung. After heparin administration, ascending aortic and bicaval venous cannulation was performed centrally through the same incision (Figure 4). Cardiopulmonary bypass with mild hypothermia (32°C) was instituted. Continuous carbon dioxide insufflation was used to facilitate the deairing procedure. A cardioplegia perfusion needle was inserted into the ascending aorta through a purse-string suture. Then, the ascending aorta was clamped, and tepid blood cardioplegia was given for myocardial protection. After the heart was arrested, right atriotomy was made. Stay sutures were put on the anterior and septal tricuspid valve leaflets for further exposure. The VSD was approached with a slight retraction of the tricuspid valve. Dacron patch and Teflon-pledgeted interrupted 5/0 Prolene sutures were used for VSD repair in all patients. In five patients with low-lying PS, a transatrial resection of the parietal and septal muscular bands was performed. The right atriotomy was closed with running sutures. In one patient who had a residual right ventricular outflow tract gradient after resection, infundibular patch augmentation was performed with glutaraldehyde-treated autologous pericardial patch. During the procedures, short-shafted tiny heartport instruments (AtriCure, Ohio, USA) were used. After weaning from cardiopulmonary bypass and decannulation, one chest tube was inserted through the pleural cavity, and a Jacson–Pratt drain was inserted into the mediastinum. The intercostal space was adapted with braided sutures, and prilocaine was used for local anesthesia. The pectoral muscle, subcutaneous tissue, and skin were closed with running sutures. While closing the thoracotomy incision, we routinely apply local anesthesia (prilocaine) to the intramuscular and subcutaneous region for post-operative pain control. The incision length was ~5-6 cm long (Figure 5).

### Data and Statistical Analysis

Statistical analysis was performed using the Web-based GraphPad software version Prism 8 (GraphPad Software Inc., CA, USA). Descriptive data were expressed in mean ± standard deviation (SD), median (min–max), or number and frequency.

## Results

There were no major complications or in-hospital mortality in any patient. All defects were repaired successfully, and no patient needed thoracotomy extension or conversion to sternotomy. The median operation time, cardiopulmonary bypass time, and aortic cross-clamp time were 157.5 min (range, 110-220 min), 81 min (range, 44-163 min), and 65 min (range, 33-131 min), respectively. The median volume of drainage was 12.78 ml/kg (range, 5.66-36.2 ml/kg), and the median volume of blood transfusion was 9.12 ml/kg (range, 0-20.4 ml/kg). No blood transfusion was required in eight patients (33.3%). The median ventilation time, intensive care unit stay, and hospital stay were 5 h (range, 2-12 h), 24 h (range, 18-160 h), and 6 days (range, 5-21 days) respectively. Atrioventricular block and infection were not observed in any patient. All patients were under echocardiographic control before discharge. One patient had a minimal (2 mm) residual left-to-right shunt. The mean follow-up time was 37 months (range, 3-78 months). All families were satisfied with the cosmetic results during follow-up. No asymmetry of the chest or the breast was noted.

### Comment

The use of minimally invasive thoracic incisions in pediatric patients aims to get better esthetic results and to reduce bleeding, infection, and post-operative complications. Various alternative incisions to standard median sternotomy have been developed to achieve the same quality of repair with cosmetically superior results. The right anterolateral and submammary thoracic incisions are widely used for treating simple congenital cardiac defects such as ASD. However, with using these approaches, problems with breast development, rib deformation, and pectoral muscle atrophy may be confronted (4). In the ministernotomy technique, peripheral cannulation is often required, and the sternotomy scar remains after surgery (5). RLMT aims to provide superior cosmetic results in the repair of congenital heart defects without changing the surgical quality. In RLMT, the incision does not have a negative effect on breast tissue development, as it does not cross the anterior axillary line. Because of the scar that remains in the bikini area, it provides much better esthetic results with great patient and parent satisfaction compared to standard sternotomy (6, 7).

The transcatheter closure of VSD has had an increasing popularity recently due to the advancement of transcatheter techniques, but especially patients with low body weight and VSD near the aortic valves are usually not suitable for this method (8). Chen et al. (9) compared percutaneous device occlusion with minimally invasive techniques for VSD closure and reported that the minimally invasive technique was more cost-effective than device occlusion but with similar complication rates.

The RLMT procedure is very similar to the standard median sternotomy technique and often does not require a special surgical instrument. All cannulations can be made centrally through the same incision, and since there is no need for peripheral cannulation, the risk of vascular complications is eliminated (10). It is also important to preserve the integrity of the sternal bone. Less mediastinal dissection results in less wound infection, less post-operative blood loss and pain, and faster recovery of the patient (11). Besides that, Li et al. (12) reported that this technique was safely and successfully used in infants weighing <5 kg. We also repaired the VSD of one patient weighing 4,600 g with the RLMT technique. According to our experience, the procedure was easier in smaller patients due to the shallower chest depth and greater skin and chest elasticity. Because the distance between the thoracotomy and the heart was longer in larger patients and the tissues were less flexible, the surgery could be a little more difficult technically, but an extra incision was never necessary in larger patients. All defects were closed over the right atriotomy; in addition, the repair of low-lying pulmonary stenosis by transatrial resection could be performed in five patients.

Liu et al. (13) performed VSD closure in 198 patients with mini-sternotomy (*n* = 66), right lateral thoracotomy (*n* = 59), and median sternotomy (*n* = 73). There was no mortality in all three groups, and the cardiopulmonary bypass and cross-clamp times were similar. Both mini-sternotomy and right lateral thoracotomy were found to be suitable for VSD closure, and a shorter duration of intensive care unit and hospital stay was an advantage of VSD closure by RLT. Wang et al. (10) performed VSD closure right vertical infraaxillary thoracotomy (RVIAT) in 274 patients, and they commented that the RVIAT can be performed with favorable cosmetic and clinical results for VSD closure. Similarly, Heinisch et al. (14) performed VSD and complete AVSD correction in 84 patients with vertical right axillary mini-thoracotomy (*n* = 25) and median sternotomy (*n* = 59). No significant differences were observed for aortic cross-clamp duration, intensive care unit stay, hospital stay, and echocardiographic follow-up. In contrast to the vertical infraaxillary incision, the RLMT incision was made parallel to the ribs, not crossing the anterior axillary line. Thus, better esthetic appearance could be provided in a way that is parallel to the body lines and remains within the bikini area.

At the beginning, we experienced some right lung injury. The right lung injuries appeared macroscopically as microhemorrhages during the surgery. In these patients, increased pulmonary opacity was observed in the post-operative chest X-ray compared to the left side. However, there was no effect on chest drainage or any pulmonary dysfunction. Although it did not affect the outcome, we suggest that heparin administration should be performed after the pericardial stay sutures were tied.

Despite the fact that our VSD series are small, we have a large experience on RMLT technique for other simpler pathologies. We think that all isolated VSDs and additional low-lying stenosis which are suitable for right atrial approach may be repaired with the RMLT technique, even in small infants.

## Conclusion

The RLMT method is a safe and effective alternative to standard median sternotomy for VSD closure and can be performed with favorable cosmetic and clinical results.

## Data Availability Statement

The original contributions presented in the study are included in the article/supplementary material, further inquiries can be directed to the corresponding author/s.

## Ethics Statement

Written informed consent was obtained from the individual(s) for the publication of any potentially identifiable images or data included in this article.

## Author's Note

Presented at: 15th Congress of Turkish Society of Cardiovascular Surgery, October 26–29, 2018, Antalya.

## Author Contributions

SA and EE contributed to conception and design of the study. BT and OG organized the database. SB performed the statistical analysis. SA wrote the first draft of the manuscript. SA, FG, and EE wrote sections of the manuscript. All authors contributed to manuscript revision, read, and approved the submitted version.

## Conflict of Interest

The authors declare that the research was conducted in the absence of any commercial or financial relationships that could be construed as a potential conflict of interest.

## Publisher's Note

All claims expressed in this article are solely those of the authors and do not necessarily represent those of their affiliated organizations, or those of the publisher, the editors and the reviewers. Any product that may be evaluated in this article, or claim that may be made by its manufacturer, is not guaranteed or endorsed by the publisher.

## References

[1] Nicholson IA Bichell DP Bacha EA del Nido PJ. Minimal sternotomy approach for congenital heart operations. Ann Thorac Surg. (2001) 71:469–72. 10.1016/S0003-4975(00)02328-6 11235691

[2] Yoshimura N Yamaguchi M Oshima Y Oka S Ootaki Y Yoshida M. Repair of atrial septal defect through a right posterolateral thoracotomy: a cosmetic approach for female patients. Ann Thorac Surg. (2001) 72:2103–5. 10.1016/S0003-4975(01)03086-7 11789801

[3] Bleiziffer S Schreiber C Burgkart R Regenfelder F Kostolny M Libera P. The influence of right anterolateral thoracotomy in prepubescent female patients on late breast development and on the incidence of scoliosis. J Thorac Cardiovasc Surg. (2004) 127:1474–80. 10.1016/j.jtcvs.2003.11.033 15116010

[4] Prêtre R Kadner A Dave H Dodge-Khatami A Bettex D Berger F. Right axillary incision: a cosmetically superior approach to repair a wide range of congenital cardiac defects. J Thorac Cardiovasc Surg. (2005) 130:277–81. 10.1016/j.jtcvs.2005.03.023 16077387

[5] Barbero-Marcial M Tanamati C Jatene MB Atik E Jatene AD. Transxiphoid approach without median sternotomy for repair of atrial septal defect. Ann Thorac Surg. (1998) 65:771–4. 10.1016/S0003-4975(97)01433-1 9527211

[6] Temur B Erek E. Repair of ventricular septal defect and pulmonary stenosis with right lateral mini-thoracotomy. Turk Gogus Kalp Dama. (2020) 28:555–6. 10.5606/tgkdc.dergisi.2020.19203 PMC7493614 32953224

[7] Kaya M Gulbeyaz SO Yildiz O Uyanik G Altin HF Kocyigit OI. Patient perception, satisfaction and cosmetic results of surgical atrial septal defect closure: Minithoracotomy versus sternotomy. Turk Gogus Kalp Dama. (2015) 23:1–8. 10.5606/tgkdc.dergisi.2015.10473

[8] Chen Q Lin ZW Hong ZN Cao H Zhang GC Chen LW. Comparison of transthoracic device closure and surgical repair with right submammary or right infra-axillary thoracotomy for perimembranous VSD. Thorac Cardiovasc Surg. (2019) 67:8–13. 10.1055/s-0038-1660809 29954030

[9] Chen ZY Lin BR Chen WH Chen Q Guo XF Chen LL. Percutaneous device occlusion and minimally invasive surgical repair for perimembraneous ventricular septal defect. Ann Thorac Surg. (2014) 97:1400–6. 10.1016/j.athoracsur.2013.12.027 24594210

[10] Wang Q Li Q Zhang J Wu Z Zhou Q Wang D. Ventricular septal defect closure using a minimal right vertical infraaxillary thoracotomy: seven-year experience in 274 patients. Ann Thorac Surg. (2010) 89:552–5. 10.1016/j.athoracsur.2009.11.026 20103340

[11] Erek E Sarioglu T Bilal MS Kinoglu B Aydemir A Sarioglu A. Sag on memealti minitorakotomisi ile “daha az invazif” kalp cerrahisi. Turk Kardiyol Dern Ars. (1999) 27:491–5. Available online at: https://jag.journalagent.com/tkd/pdfs/TKDA_27_7_491_495.pdf

[12] Li G Su J Fan X Li Z Zhang J Zhu Y. Safety and efficacy of ventricular septal defect repair using a cosmetic shorter right lateral thoracotomy on infants weighing less than 5 kg. Heart Lung Circ. (2015) 24:898–904. 10.1016/j.hlc.2015.02.010 25769663

[13] Liu H Wang Z Xia J Hu R Wu Z Hu X. Evaluation of different minimally invasive techniques in surgical treatment for ventricular septal defect. Heart Lung Circ. (2018) 27:365–70. 10.1016/j.hlc.2017.01.014 29153964

[14] Heinisch PP Wildbolz M Beck MJ Bartkevics M Gahl B Eberle B. Vertical right axillary mini-thoracotomy for correction of ventricular septal defects and complete atrioventricular septal defects. Ann Thorac Surg. (2018) 106:1220–8. 10.1016/j.athoracsur.2018.05.003 29859151

